# Comparison of safety and number of post-operative visits of patients in convenient day versus conventional first day follow-up after phacoemulsification

**DOI:** 10.12669/pjms.37.5.4121

**Published:** 2021

**Authors:** Zahid Kamal, Ahmad Zeeshan Jamil, Hira Shuja Khokhar, Farah Huma

**Affiliations:** 1 Zahid Kamal, MBBS, FRCS(Ed), FCPS, FRVEEH, MCPS (HPE). Professor of Ophthalmology, Sahiwal Medical College, Sahiwal, Pakistan; 2 Ahmad Zeeshan Jamil, MBBS, MCPS, FCPS, FRCS, FCPS (VRO). Associate Professor of Ophthalmology, Sahiwal Medical College, Sahiwal, Pakistan; 3 Hira Shuja Khokhar, MBBS. Post Graduate Trainee, Department of Ophthalmology, Unit 1, Mayo Hospital Lahore, Pakistan; 4 Farah Huma, MBBS. Post Graduate Trainee, Department of Ophthalmology, Unit 1, Mayo Hospital Lahore, Pakistan

**Keywords:** Cataract, Cost, Intraocular lens, Phacoemulsification, Safety

## Abstract

**Objectives:**

To compare safety and number of post-operative visits of patients in convenient day versus conventional first day follow-up after phacoemulsification

**Methods:**

This observational cohort study was conducted in Department of ophthalmology, Sahiwal Medical College, Sahiwal from November 2019 to August 2020. There were 600 patients who underwent uncomplicated phacoemulsification with intraocular lens implantation. Patients were allocated into two groups. Group-I comprised of patients with convenient day follow-up during the first post-operative week. Group-II comprised of the patients with conventional first day follow-up. Rate of complications, number of visits during the first month and final visual acuity were recorded.

**Results:**

In Group-I post-operative complications were noted in 12.67% cases on first follow up visit and in 2.67% cases on first month follow up visit. In Group-II post-operative complication were noted in 22 % cases on first follow up visit and in 4% cases on first month follow up visit. Common postoperative complications were corneal oedema, anterior segment intraocular inflammation, residual lens matter in anterior chamber and intraocular lens subluxation. There was no difference in presenting and postoperative visual acuity between the two groups. Mean follow-up visits were 2.23 ± 0.42 in Group-I and 3.55 ± 0.50 in Group-II.

**Conclusion:**

Convenient day follow-up is as safe as conventional first day follow-up. Convenient day follow-up significantly reduces the number of post-operative visits. This would translate into cost reduction both for the patients and the health care facility.

## INTRODUCTION

Cataract is the leading cause of blindness.^1^ In the developing countries burden of cataract is far more than its impact on the patient’s health. It severely affects the quality of life, economic growth, and academic progress. There are many barriers in the control of preventable blindness caused by cataracts. These include poverty, ignorance, and lack of health care services.

In Pakistan incidence of blindness due to cataract was 1.78% in 1989-1990 and it fell to 0.9% in 2002-2004. Collaborative efforts in the control of preventable blindness due to cataract are expected to result in further reduction in the incidence of blindness.^2^

Cataract surgery by phacoemulsification with intraocular lens implantation has become the standard of care all over the world. Cataract surgeries are mostly performed on day care basis. It has resulted in cost reduction of the procedure without compromising the visual outcome.^3^ Patient’s satisfaction is higher in day care surgical procedure as compared to the inpatient surgical approach. There has been no difference in the safety profile of day care surgery as compared to the inpatient surgery.^4^ Day care surgery cuts the expenditure of the patient and the health care facility.^5^ Patient time and funds are spared and there is less burden over the resources of health care delivery system.^6^

After uneventful cataract surgeries patients have to come for follow-up. In developing countries patients have to struggle with bad roads. They have to bear miserable transportation facilities. Expenditure during travel to hospitals put extra burden on patients. Moreover, follow-up patients put extra load on already struggling health care system. So, it is important to cut the number of follow-up visits to as less as possible without compromising the safety. Studies have shown safety of first follow up visit during the first two weeks instead of coming at first day.^7^ Patient were found to be satisfied with first follow up visit at convenient day.^8^ Less number of visits would reduce the stress on the patients and the surgeons. It would reduce the cost associated with extra follow up visits. Instead of first post-operative day follow-up, follow-up on a day that is convenient to both the patient and the surgeon would reduce the number of follow-up visits. Purpose of the current study was to compare safety and number of post-operative visits of patients in convenient day versus conventional first day follow-up after phacoemulsification.

## METHODS

This observational cohort study was conducted in ophthalmology department of District headquarter teaching hospital affiliated with Sahiwal Medical College, Sahiwal from November 2019 to August 2020. Institutional review board (IRB) approval was sought before the commencement of the study. IRB approval number was 76/DME/SLMC/SWL, dated 22/10/2019. All the patients were asked to sign an informed consent. Inclusion criteria included cataract in the eye to be operated. Types of cataract were nuclear sclerosis, posterior sub capsular, cortical, posterior polar and mature. Age range was from 40 to 75 years and both genders were included. Exclusion criteria included previous history of eye trauma or surgery, glaucoma, corneal opacity, intraocular inflammation, physical and mental limitations that may prevent adherence to post-operative care. Complications occurring during surgery resulted in the exclusion of patients from the study. Those complications were posterior capsular rupture, lens drop into the vitreous, vitreous prolapse, zonular dehiscence and surgical incision leak requiring stitching. Those patients who did not come for follow-up were also excluded from the study. All the patients were examined on slit lamp thoroughly. Detailed anterior and posterior segment examination was done. All the patients were operated by a single surgeon and foldable intraocular lens was implanted. Patients were divided into two groups using random number tables. Group-I/cohort group, patients were advised for a convenient day post-operative follow-up during the first week after cataract surgery. This convenient day was decided by the mutual understanding of the patients and the surgeon. After that Group-I patient were advised to come for post-operative follow-up visit at day 30. Any unscheduled visits during the first month post operatively were recorded. Group-II (Control) patients were advised for first post-operative day follow-up. After that they were advised to come at day seven and day 30 post-operative follow-up. Any unscheduled visits during the first month post operatively were recorded.

At each follow-up visit detailed slit lamp examination was performed. Record of any complication was made. Intraocular pressure was measured. Best corrected visual acuity was also noted.

### In all the patients following end point indicators were recorded:

1. Number of patients’ follow-up visits
2. Best corrected vision at the end of one month
3. Complications at first visit and at one month

All the patients were given same postoperative care and instructed to come to the hospital in case of any emergency. All the patients were advised to instill moxifloxacin 0.5% and dexamethasone 0.1% combination eye drops. During the first week frequency of eye drops was two hourly during the waking hours. After that eye drops were given four times in a day for next three weeks in both groups.

All data was analyzed by SPSS version 23. Frequencies of qualitative variables like gender, best correct visual acuity and complications were calculated. Mean and standard deviation of quantitative variables like age and number of patients follow up visits were calculated.

## RESULTS

There were 300 patients in each group. Mean age of the patients was 57.25±10.03 years in Group-I and 57.53±9.83 years in Group-II. In Group-I/cohort group, there were 146 (48.67%) male and 154 (51.33%) females. While in Group-II/control group, there were 144 (48%) males and 156 (52%) females. Presenting and final visual acuity at the end of one month are shown in Table-I. There was no difference in presenting and postoperative visual acuity between the two groups. Chi-square value for preoperative visual acuity between the two group is 0.15 with p value being 0.93. Chi-square value for postoperative visual acuity between the two group is 1.25 with p value being 0.54.

**Table-I:** Preoperative and postoperative visual acuity N=300.

Visual acuity	Group-I (cohort)		Group-II (control)	

	Pre-operative	Post-operative	Pre-operative	Post-operative
6/9 to 6/18	24(8%)	284(94.67%)	26(8.67%)	282(94%)
6/24 to 6/60	156(52%)	16(5.33%)	152(50.67%)	18(6%)
Less than 6/60	120(40%)	0	122(40.67%)	0

Key: N= number of cases in each group

Group-I = convenient day follow-up, Group-II= follow-up at first post-operative day.

Most common variety of cataract was nuclear sclerosis cataract. Distribution of cases according to the variety of cataract is given in Table-II.

**Table-II:** Type of cataract N=300.

Type of cataract	Group-I/cohort	Group-II/control
Nuclear sclerosis	176 (58.67%)	178 (59.33%)
Cortical	62 (20.67%)	50 (16.67%)
Posterior sub capsular	54 (18%)	40 (13.33%)
Mature	8 (2.67%)	24 (8%)
Posterior Polar	0	8 (2.67%)

Key: N= number of cases in each group.

Group-I= convenient day follow-up,

Group-II= first post-operative day follow-up.

In Group-I/cohort group, post-operative complications were noted in 12.67% cases on first follow-up visit and in 2.67% cases on first month follow-up visit. In Group-II/control group, post-operative complications were noted in 22% cases on first follow-up visit and in 4% cases on first month follow-up visit. There was statistically significant difference in the rate of complications noted at first follow-up visit between the two groups with p value being 0.04. At one month follow-up visit there was no difference in frequency of complication between the two groups with a p value of 0.13. Post-operative complications in both groups are presented in Table-III.

**Table-III:** Postoperative Complications. N=300.

Complications	Group-I/cohort		Group-II/control	

	First follow up	First month follow up	First follow up	First month follow up
Corneal oedema	10 (3.33%)	0	20 (6.67%)	
Anterior chamber reaction	20 (6.67%)	8 (2.67%)	30 (10%)	8 (2.67%)
IOL subluxation	4 (1.33%)	0	8 (2.67%)	
Lens matter in anterior chamber	4 (1.33%)	0	8 (2.67%)	4 (1.33%)

Key: N= number of cases in each group

Group-I= convenient day follow-up, Group-II= first post-operative day follow-up.

During the first week follow-up period more patients came for unscheduled follow-up visits in Group-II/control as compared to Group-I/control. Number of patients who came for unscheduled follow-up visits in both groups is given in Table-IV.

**Table-IV:** Unscheduled follow-up visits N=300.

Unscheduled follow-up	Group-I/cohort	Group-II/control	Chi square
First week	18(6%)	44(14.67%)	12.16 p=0.000
Second week	16(5.33%)	60(20%)	29.16 p=0.000
Third week	26(8.67%)	42(14%)	4.25 p=0.04
Fourth week	8(2.67%)	20(6.67%)	5.40 p=0.02

Key:N= number of cases in each group,

Group-I= convenient day follow-up, Group-II= first post-operative day follow-up.

Mean follow-up visits in Group-I were 2.23 ± 0.42 and 3.55 ± 0.50 in Group-II. There was statistically significant difference in the means of two groups with p value of 0.000.

## DISCUSSION

Cataract is the opacification of the clear crystalline lens. Age related cataract is the most common cause of visual impairment.^9^ Cataract impairs a person’s ability to take part in day-to-day activity of life. Phacoemulsification has become the standard of care in the management of visually significant cataract.^10^ With the implantation of foldable intra ocular lens, it further fastens the rehabilitation of the patient. It makes the procedure a day care procedure in its true sense.^11^

In the current study, at the end of one month best corrected visual acuity was comparable in both groups. Same results have been observed by the Tinley and coauthors.^12^ In their study, review of patients at two weeks after cataract surgery as compared to next day review was not associated with increased risk of complications. Moreover, there was no difference in postoperative visual acuity between next day review group and 2 weeks review group.

Complications encountered in both groups were comparable. At first follow-up visit, complications in Group-II/control group were more than in Group-1/cohort group. Those complications were corneal oedema and anterior chamber reaction. Plausible explanation for that might be convenient day follow-up group patient having time to settle those early postoperative complications before they came for follow-up. At the end of one month both group patients did well, and complications settled.

It is safe not to see the operated patient on the very next day. Serious post cataract surgery complications like post-operative endophthalmitis and retinal detachment are rare. These complications are seldom seen on the very first day after cataract surgery.^5^,^13^,^14^ Post cataract surgery telephonic conversation with the patient is found to be safe and satisfying to the patient.^7^,^8^ Patient’s first follow-up visit to the health care facility can be safely prolonged up to two weeks.^15^ In the current study convenient day postoperative follow-up was comparable to the conventional first day follow-up in terms of safety. Our results are supported by the work of Ahmed and co-authors.^16^ They found cataract surgery patient can be safely reviewed after four days to detect and manage postoperative complication without compromising patient’s care.

Post cataract surgery first day follow-up is not without advantages. These include opportunity to early detect any post-operative complication, reassurance of the patients regarding any concerns, instructions to the patient about proper use of medicines and eye care.^6^,^17^ On the other hand follow-up on convenient day during the first post-operative week has its advantages. Patients and surgeons feel convenient, number of visits to health care facility are reduced, patient’s time and money is spared and there is less burden over the health care facility.^18^ In the current study number of unscheduled follow-up visits were more in control group as compared to the cohort group. During the first two weeks this difference was statistically significant. Possible explanation to this fact might be difference in patients counseling in two groups.

In low and middle income countries most of the visual impairment is attributed to the cataract. While in high income countries cataract accounts for a small proportion of visual deterioration.^19^-^21^ Day care cataract surgery is a practical measure to deal with shortage of inpatient health care facilities in countries like Pakistan. Less resources are consumed in day care surgery as compared to the inpatient care. Going one step further, convenient day follow-up after day care cataract surgery further reduces the cost of cataract surgery. In this way cataract surgery would be utilizing less resources. Resources would be directed to other demanding eye surgeries.

In the current study convenient day postoperative follow-up was comparable to the conventional first day follow-up in terms of safety.

### Limitation of the Study

It include relatively small sample size. Other limitation include study is based on a single tertiary care hospital. Moreover, satisfaction level of patients and surgeon should be assessed with convenient day follow-up after cataract surgery. Nevertheless, the present study found safety of convenient day follow-up comparable with conventional first day follow-up without compromising patient’s care.

## CONCLUSION

Convenient day follow-up visit can replace the conventional first day follow-up visit. Convenient day follow-up is as safe as conventional first day follow-up. There are no additional risks associated with convenient day follow-up. It significantly reduces the number of post-operative visits. This would translate into cost reduction both for the patients and the health care facility.

### Author’s Contribution:

**ZK:** Concept and Design of the study, Final approval of manuscript

**AZJ:** Writing of article, Data Collection. Responsible and accountable for integrity of the work.

**HSK:** Statistical analysis, Literature search.

**FH:** Literature search, Critical Review.
